# Back to youth: flipping the switch on tissue-resident macrophages

**DOI:** 10.1038/s41392-026-03068-7

**Published:** 2026-10-12

**Authors:** Priya Veluswamy, Jens Wippermann, Max Wacker

**Affiliations:** Heart Surgery Research, Department of Cardiothoracic Surgery, Medical Faculty of Otto-von-Guericke University, Magdeburg, Germany

**Keywords:** Senescence, Inflammation

A recent study published in *Science*^1^ deciphered the mechanistic role of the tissue-resident macrophage (TRM)-E-prostanoid receptor 2 (EP2) axis in preserving a youthful physiological state across multiple organs. This study highlights the targeted modulation of EP2 signaling in TRMs, through either genetic ablation or pharmacological antagonism, as a mechanism for clearing tissue-accumulated senescent neutrophils and thereby restoring age-related structural and functional decline across systemic and multiorgan phenotypes towards a more youthful state.

TRMs are tissue-resident, self-renewing phagocytes that maintain tissue homeostasis by clearing cellular debris. TRMs express four prostaglandin E2 (PGE2) receptors (EP1–EP4), with EP2 signaling exerting context-dependent pro- and anti-inflammatory effects.^2,3^ Despite these immunomodulatory properties, the contribution of the TRM-EP2 axis to organ aging remains unexplored.

Tan and colleagues^1^ investigated the role of the TRM–EP2 axis in organ aging using tamoxifen-inducible, EP2-deficient mice (EP2 cKO), generated by crossing *Cx3cr1*^creER^ (CX3C motif chemokine receptor-1) with *Ptger2*^lox/lox^ (prostaglandin E receptor 2) mice. Further, T-cell immunoglobulin and mucin domain-containing protein-4 (TIM-4) -based stratification and tissue-specific markers supported that *Cx3cr1*^creER^-mediated EP2 deletion reflected macrophage residency rather than ontogeny. EP2 function was assessed in young (6–8 months) and aged (23–25 months) mice, with age-matched EP2-sufficient littermates as WT controls.

Given that aging is associated with chronic, low-grade inflammation („inflammaging“),^4^ the authors assessed inflammatory signatures across the liver, lungs, hippocampus, heart, kidney, colon, and plasma. Aged EP2-sufficient WT mice displayed a pronounced inflammaging, marked by elevated pro-inflammatory cytokines (tumor necrosis factor (TNF)-α, interleukin (IL)-6, and IL-1β), type I and II interferon responses, and elevated chemokines. These age-associated immune signatures were markedly attenuated in EP2-cKO mice, approaching youthful levels. Extending these observations to organ-specific outcomes, EP2 deletion mitigated age-associated functional decline, as evidenced by (i) improved cognitive functions (spatial and recognition memory); (ii) reduced subcutaneous and visceral adiposity; (iii) increased muscle mass, forelimb strength, and preserved muscle fibers; (iv) restored muscle regeneration and myonuclear organization; (v) improved cardiac functions, including preserved left ventricular ejection fraction, normalized ventricular geometry and reduced cardiac fibrosis by decreased collagen accumulation; (vi) restored gut barrier integrity and youthful endotoxin levels, and (vii) reduced liver-associated inflammation as evidenced by youthful levels of liver enzymes and C-reactive protein (CRP). Collectively, these findings identify EP2 signaling as a key driver of systemic inflammaging and age-related multiorgan dysfunction.

Further high-throughput plasma proteomics revealed age-related suppression of liver X receptor/retinoid X receptor (LXR/RXR) and integrin signalings, acute-phase responses, neutrophil degranulation, and glycolytic pathways, all of which were restored toward youthful levels in EP2 cKO mice. Tissue-resolved analyses further implicated liver cells as major contributors to these systemic proteomic changes. Consistently, liver TRMs showed a selective age-associated decline accompanied by impaired mitochondrial mass and membrane potential and increased superoxide production. These defects were largely reversed in aged EP2 cKO mice, implicating EP2 signaling as critical regulator of mitochondrial homeostasis and liver TRM maintenance during aging.

Focusing on the liver, which exhibited dynamic changes in aging-related inflammatory signatures, the authors identified four distinct hepatic neutrophil trajectories (Neu 1 to Neu 4). Given that cellular senescence is a hallmark of aging^5^ and a driver of chronic inflammation, the senescent Neu 2 and apoptotic Neu 3 subsets predominated in aged WT mice, whereas Neu 1 and Neu 4 were more prominent in young EP2-sufficient mice. Flow cytometry confirmed accumulation of C-X-C chemokine receptor type-4 (CXCR4)^hi^ (senescent) and Annexin V^+^ (apoptotic) neutrophils in aged WT livers, implicating impaired TRM-mediated efferocytosis, particularly of senescent neutrophils. Persistence of hepatic CXCR4^hi^ neutrophils contributed to tissue injury through increased myeloperoxidase (MPO) activity. Furthermore, senescence-associated secretory protein (SASP)-mediated paracrine signaling in the liver parenchyma induced hepatocellular intracellular adhesion molecules (ICAM)-1 upregulation, indicative of inflammatory stress. Although aging broadly impairs efferocytosis, senescent neutrophils in aged WT mice show increased dependence on integrin-mediated TRM stabilization. Though elevated lymphocyte function-associated antigen 1 (LFA-1) appears compensatory rather than functionally adhesive, reduced integrin-activating cofactors destabilize the TRM–neutrophil axis, contributing to defective efferocytosis as a key mechanism. Consistent with defective engulfment, aged WT mice exhibited a dysregulated post-engulfment program, with reduced pro-engulfment factors and increased inhibitory regulators.

**Fig. 1:**
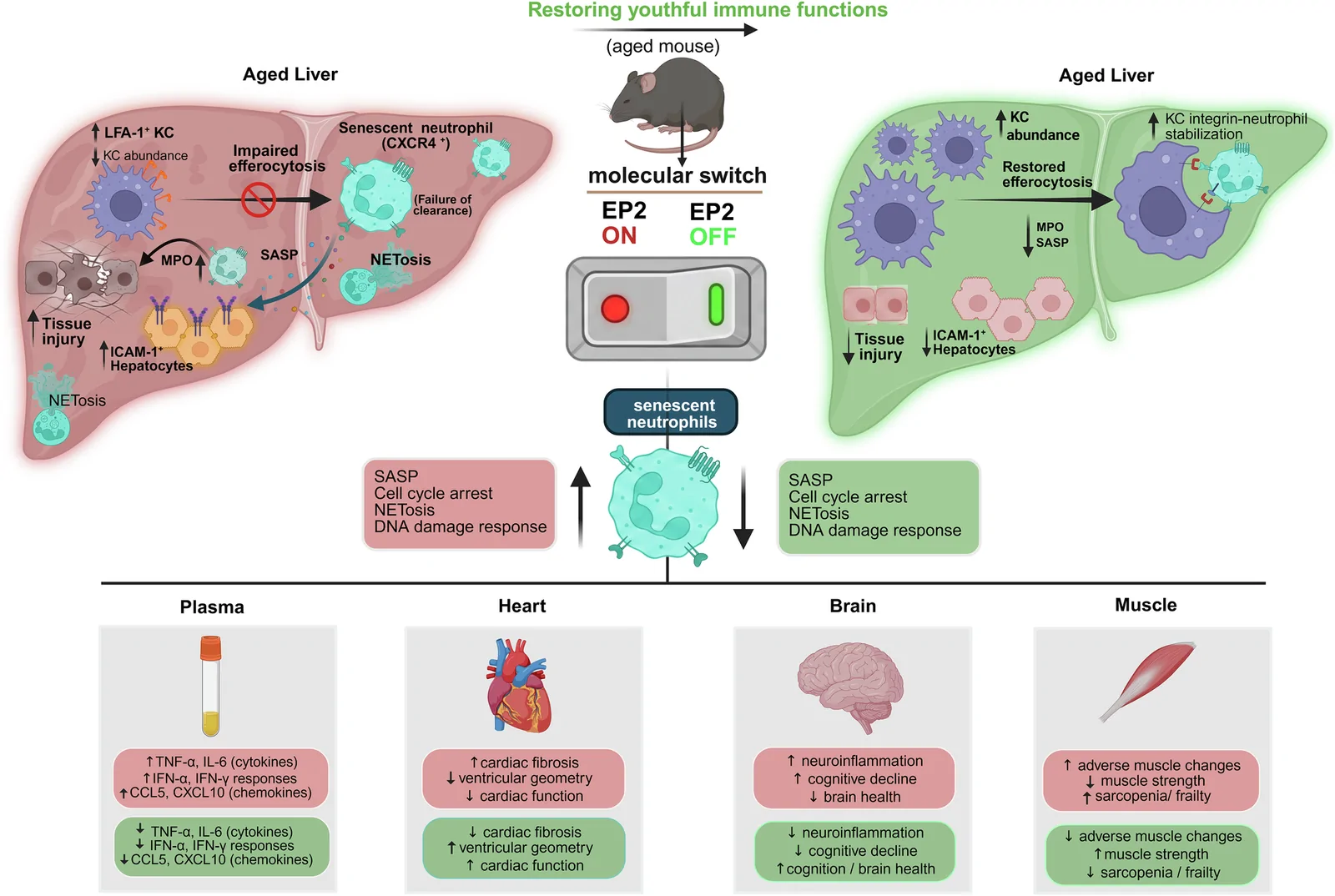
Restoring youthful immune functions by switching off E-prostanoid receptor 2 (EP2) on tissue-resident macrophages. **EP2 ON** (left schematic, with intact EP2 signaling) in the aged liver is characterized by dysregulated LFA-1^+^ Kupffer cell (KCs) and reduced KC abundance. This leads to impaired clearance of CXCR4^+^ senescent neutrophils and defective efferocytosis. Persistent senescent neutrophils promote liver injury through increased myeloperoxidase (MPO) activity. The senescence-associated secretory phenotype (SASP) produced by senescent neutrophils exerts paracrine effects that increases intracellular adhesion molecules (ICAM-1) expression on hepatocytes under inflammatory stress. **EP2 OFF** (right schematic, with disabled EP2 signaling) in aged liver restores KC abundance and integrin expression, thereby stabilizing senescent neutrophils and promoting efficient efferocytosis. The restored clearance of senescent neutrophils is characterized by reduced MPO activity and SASP production, resulting in diminished liver injury and lower ICAM-1 expression on hepatocytes. Beyond the liver, **EP2 OFF** mice exhibit broader improvements in extrahepatic organs compared with **EP2 ON** mice, including (i) normalization of elevated circulating plasma levels of pro-inflammatory cytokines, interferon responses, and chemokines; (ii) attenuation of cardiac fibrosis, accompanied by improved cardiac geometry and function; (iii) reduced neuroinflammation accompanied by improved cognitive performance and preserved brain health; and (iv) attenuation of age-associated muscle alterations, including improved strength, and reduced measures of sarcopenia and frailty. Collectively, these findings support the conclusion that EP2-deficiency preserves TRM-mediated efferocytosis and clearance of dysfunctional neutrophils during aging, thereby limiting neutrophil-driven hepatic inflammation and injury. This schematic figure was created using BioRender.com

Strikingly, EP2 deficiency substantially improved age-associated neutrophil accumulation, TRM–neutrophil disruption, and impaired efferocytosis, restoring the hepatic neutrophil–TRM axis toward a more youthful state. Collectively, these findings support that EP2 deficiency preserves TRM-mediated efferocytosis and clearance of dysfunctional neutrophils during aging, thereby limiting neutrophil-driven hepatic inflammation and injury (Fig. 1).

To corroborate these genetic knockout findings, a pharmacological EP2 antagonist was employed that normalized circulating and hepatic senescent neutrophils to youthful levels, restored KC abundance, reduced LFA-1^+^ KC, and enhanced senescent neutrophil uptake by KCs. Extending these findings to human aging and disease, atlas-based analyses revealed reduced TRM proportions in aged and diseased liver and heart, accompanied by increased neutrophils abundance specifically in aged and diseased liver, supporting the liver as a primary site for neutrophil efferocytosis. Although the EP2-encoding gene (*PTGER2)* was not significantly associated with longevity in published genome-wide association studies (GWAS), genes involved in cell-cycle regulation, cellular senescence, and immune functions were significantly implicated, further supporting the broader relevance of TRM/EP2 axis in aging.

Overall, Tan et al. excellently position TRMs as active regulators of organismal aging rather than passive bystanders, highlighting impaired immune-mediated clearance as a determinant of senescent-cell burden alongside increased senescent-cell generation. This is particularly relevant to neutrophil senescence, an underappreciated and dynamic component of inflammaging, traditionally overlooked because of neutrophil short-lived nature. Their accumulation likely reflects both prolonged survival and defective clearance, and resolution of inflammation. Rather than eliminating senescent neutrophils, EP2 receptor inhibition in TRMs restores their endogenous recognition and clearance program. Although the study centers on the liver, its phenotypic effects extend across organs, suggesting that impaired hepatic immune surveillance may contribute to systemic inflammaging. Collectively, these findings identify EP2 on TRM as a molecular target for restoring youthful immune functions. From a broader perspective, these findings raise questions about whether EP2-dependent efferocytosis defects in aging extend across tissue-resident macrophages of different developmental origins and finally, how pharmacological EP2 antagonism restores hepatic neutrophil clearance and translates into clinically-relevant systemic benefits.
